# Therapeutic Targeting of Neutrophil Extracellular Traps Improves Primary and Secondary Intention Wound Healing in Mice

**DOI:** 10.3389/fimmu.2021.614347

**Published:** 2021-02-25

**Authors:** Annika Heuer, Carolin Stiel, Julia Elrod, Ingo Königs, Deirdre Vincent, Patrick Schlegel, Magdalena Trochimiuk, Birgit Appl, Konrad Reinshagen, Laia Pagerols Raluy, Michael Boettcher

**Affiliations:** ^1^ Department of Pediatric Surgery, University Medical Center Hamburg-Eppendorf, Hamburg, Germany; ^2^ Children’s Medical Research Institute, Sydney University, Westmead, NSW, Australia

**Keywords:** scars, burns, wound healing, neutrophil extracellular traps, DNases

## Abstract

**Background:**

Neutrophils are the first responders in wound healing after injury that mediate pro- and anti-inflammatory activities i.a. through the formation of extracellular traps (NETs). However, excessive NETs presence in wound tissue can cause local hyperinflammation and -coagulation resulting in delayed wound healing. To improve wound healing, we aimed to examine the role of NETs and DNase1 on primary and secondary wound healing.

**Methods:**

The study included 93 C57BL/6 mice, with 3 different genotypes: wildtype, Pad4-, and DNase1-Knockout (KO). Pad4-KO mice show limited NETs formation, while DNase1-KO mice cannot disintegrate them. All 3 genotypes were included in (1) a laparotomy group and (2) a thermal injury group. Animals in both groups either received DNase1 or a vehicle i.p. post wound induction and wound assessment and euthanasia were conducted. Laparotomy and burn scars were assessed using the stony brook scar evaluation scale and modified Yeong scale respectively. Tissue was analyzed histologically using H&E staining. Ly6g, Collagen I and III, SMA, and Fibrinogen were visualized and neutrophils activation (NE, MPO) and NETs (H3cit) formation assessed.

**Results:**

All animals survived with no complications. DNase1 treatment led to a significantly improved scar appearance in both groups, which was also seen in Pad4-KO mice. In the laparotomy group DNase1 improved collagen deposition and fibrin concentration was significantly reduced by DNase1 treatment. Markers of neutrophil activation were significantly reduced in the treatment and Pad4-KO group. In the thermal injury group wound closure time was significantly reduced after DNase1 treatment and in the Pad4-KO group. Even though inflammation remained high in the thermal injury model over time, neutrophil activation and NETs formation were significantly reduced by DNase1 treatment compared to controls.

**Discussion:**

Primary and secondary intention wound healing is improved by targeting NETs through DNase1 treatment or genetic KO, as assessed by wound closure time and scar appearances. Additionally, wound stability was not affected by DNASE treatment. The results suggest that overall wound healing is accelerated and DNase1 appears to be a promising option to reduce scar formation; which should be evaluated in humans.

## Background

After an injury our innate immune system reacts within hours and days through an array of mechanisms which can exhibit both pro- and anti-inflammatory activities (1). A delicate equilibrium is formed that, once interrupted, can tilt from protecting the host to mediating hyperinflammation, further injury, and increasing mortality (1). Neutrophils are the most abundant cell type in the circulatory system, are regarded as the first line of defense in the innate immune system, and constitute the main leukocytes involved in the early phase of wound healing (2, 3). The short half-life of neutrophils in circulation, which is approximately 4 h, is balanced by their continuous and tightly controlled release from the bone marrow. However, recent studies have shown that neutrophils may differentiate into distinct subsets defined by specific phenotypes and functional profiles (4). As such, neutrophiles can reverse transmigration and reenter the circulatory system after shifting their phenotype towards a proinflammatory state with a longer life span of about 5.4 days, leading to severe systemic inflammation (5).

In response to infection and injury, neutrophils form extracellular traps (NETs), consisting of a tight network of nuclear material, lined with cytotoxic proteins such as myeloperoxidase (MPO) and neutrophil elastase (NE) (6, 7). NET formation is part of an evolutionarily conserved innate immune response that is directed at capturing and killing microbial pathogens either through (1) a programmed cell death pathway or (2) through discharging parts or their whole nucleus in a non-lytic matter (6–9). In spite of their antimicrobial properties, the excessive presence of NETs can be detrimental to the host in some cases, and hence NETs have been aptly acknowledged as “double-edged swords of innate immunity” involved in both stimulating and resolving inflammation (10, 11). NETs formation is not only induced by pathogens, it is also stimulated through endogenous danger signals and can further modulate the immune response though priming other cells to induce sterile inflammation. NETs may also stimulate platelet adhesion, resulting in activation of the coagulation cascade causing deleterious effects (6, 12–15). In fact, it has been shown that disturbed interactions, excessive release of NETs into the circulation, and an overexpression of cytokines contribute significantly to the pathology of several inflammatory conditions, such as autoimmune diseases, sepsis, ischemia reperfusion injury, thrombosis, endothelial damage, and hyperinflammation (16–19).

Different factors can interfere with the wound healing process, leading to an impairment of the physiological wound healing; resulting in (1) delayed acute wounds, (2) chronic wounds, or (3) excessive scar formation, causing a tremendous psychosocial burden to afflicted patients (20–22). Additionally, it has been reported that nonhealing wounds result in enormous health care expenses with costs being estimated at more than $3 billion per year in the US (22). Although inflammation is indispensable for wound healing, it is closely associated with scar formation (21, 23, 24). In this context pathologic scars are assumed to be the result of hyper- or chronic inflammation of the reticular dermis (23).

Inflammation appears to be of particular importance in the context of thermal injuries. In burns, early debridement is essential in order to limit the inflammatory cascade, cease the hypermetabolic state, and limit secondary damage, since the removed eschar constitutes an inflammatory matrix and moreover presents an ideal breeding ground for pathogens (25–27). Furthermore, early re-epithelialization is one of the most important positive prognostic markers for an optimal outcome after thermal injury. A significant proportion of the tissue loss can be caused by secondary expansion of necrosis into the surrounding initially vital neighboring dermis, leading to an increased burn depth and area (28). Neutrophils infiltrate the wound after burn trauma and mediate microvascular damage in the zone of stasis through the formation of NETs (29). Hence, this process might be the result of neutrophils triggering local inflammatory responses and NET induced hypercoagulation at the burn wound site.

Activated neutrophils and elevated NET levels can be found in the adjacent tissue up to 60 days after the initial thermal injury in pigs and human, where the activated neutrophils produce large quantities of proteases and matrix metalloproteinases (30, 31). This results in prolonged inflammation, increased tissue damage, and delayed wound healing, which in turn promotes formation of hypertrophic scars (19, 32). Consequentially, neutrophils and induction of NET formation should be tightly regulated during the inflammatory phase of both primary and secondary intention wound healing to prevent excessive tissue loss, which can spread out far beyond the initially affected area. Recently, DNases have been reported to counteract local hypercoagulability and clotting-induced hypoxia, by dissolution of NETs formation, resulting in significantly enhances tissue perfusion and accelerated wound healing (7, 13, 14, 33). In the absence of DNASES, intravascular NETs form aggregates (NET clots) that can occlude blood vessels and cause ischemic end organ failure damage during inflammatory responses (33, 34). Thus, as clotting and inflammation processes are essential for wound healing, the aim of this study is to examine the role of NETs and DNase1 on primary and secondary wound healing.

## Materials and Methods

### Study Design

The study was approved by the Hamburg State Administration for animal research (73/15, 63/16). A total of 93 six- to eight-week-old mice (C57BL/6) were utilized for the two experimental models. Mice with a Pad4- or DNase1-Knockout with the same genetic background (C57BL/6) were used to examine the role of NETs and DNase1 in the process of wound healing. The DNase1-KO mice were generated as described earlier (33, 35). The WT mice and the Pad4-KO mice were obtained from Jackson Laboratory. PAD4 is a histone-modifying enzyme that is essential for NETs formation and its inhibition has been shown to limit NETs formation (36). For comparison, DNase1-Knockout mice were also employed. As DNase1 is known to disintegrate NETs (7), DNase1-Knockout mice are incapable of NET-resolution. All environmental parameters within the animal facility complied with the German guide for the care and use of laboratory animals (Tierschutzgesetz).

### Animal Procedures

The mice were randomly divided into various groups. For better standardization all interventions were performed by the same operator. Anesthesia was induced with 5% isoflurane gas (Baxter, Unterschleißheim, Germany) and maintained with 2.5% isoflurane gas delivered through a facemask. Preoperative antisepsis was performed with Octenisept.

### Model 1: Laparotomy

**Figure 1:**
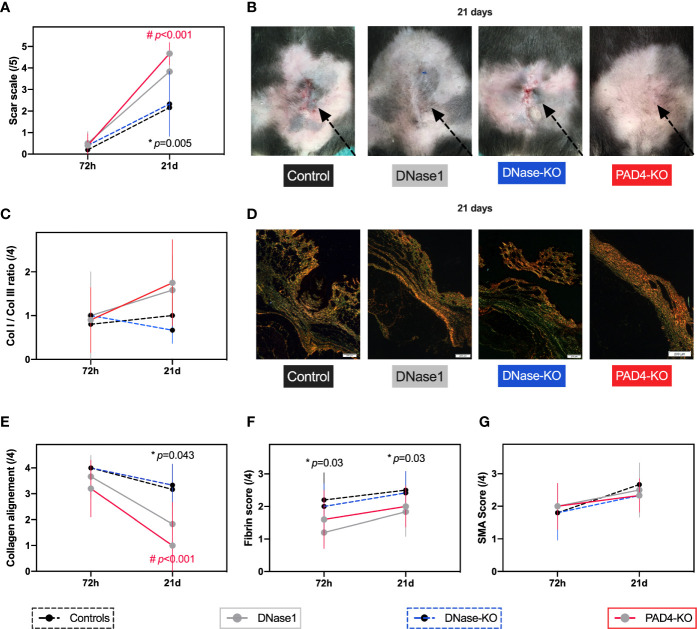
Therapeutic targeting of NETs improves primary intention wound healing. In all animals a 2.5 cm long laparotomy was performed. For the wildtype and knockout mice two timepoints (72 h, 21 days) were performed. Controls n=6, DNase1 n=6, DNase1-KO n=5, Pad4-KO n=6. **(A, B)** Animals that received DNase1 had significantly superior scar scores than controls. In mice that were unable to produce NETs (Pad4-KO) a similar effect was found. **(C–E)** Animals with DNase1 treatment or animals without NETs showed a significantly faster switch from collagen 3 to 1 and better collagen alignment indicating a faster maturation of the scar. **(F)** As previously reported DNase1 reduced Fibrin levels in the scar. **(G)** SMA was not affected by DNase1 treatment or in animals without NETs. Data shown as Mean ± SD. Comparison was performed always in comparison with controls. Statistics: mixed-effect model with Geisser-Greenhouse correction as well as Dunnett’s multiple comparison test. *DNase1 vs. controls. ^#^PAD4-KO vs. controls.

Primary intention wound healing was induced *via* median laparotomy using scissors (2.5 cm length) followed by a single-layer continuous suture (Prolene 5-0; Ethicon, Norderstedt, Germany) in all animals. No suture removal was performed. Mice in the treatment group received DNase1 (Pulmozyme, Roche, Mannheim, Germany) with a dosage of 10 mg/kg body weight *via* i.p. route for 72 h every 12 h as shown **Figure 1B**. Control animals received a vehicle with the same treatment intervals as the case group. Euthanasia was performed after 72 h or 21 days *via* decapitation under general anesthetic with Isoflurane. Experimental design and treatment strategy are summarized in **Supplement 1**.

### Model 2: Thermal Injury

Thermal injuries, serving as a model for secondary intention wound healing, were induced as described previously (37, 38). In short, a 1.5 cm x 1.5 cm large burn injury was induced. After discontinuation of anesthesia, all animals were housed in the animal facility. Again, animals in the treatment group received DNase1 (Pulmozyme, Roche, Mannheim, Germany) with a dosage of 10 mg/kg body weight *via* i.p. route for 7 days every 12 h. Control animals received a vehicle with treatment intervals. Animals were euthanatized after 72 h, 7 days, 14 days, or 28 days as described above. Experimental design and treatment strategy are summarized in **Supplement 1**.

### Scar Assessment

Scar assessment of model 1 (laparotomy wounds) were evaluated by two surgeons, blinded for the treatment group, before euthanasia using the Stony Brook Scar Evaluation Scale (39). This five-item ordinal wound evaluation scale incorporates assessments of five distinct attributes (width, height, color, suture marks, and overall appearance) with a binary response (1 or 0), resulting in a total score ranging from 0 (*worst*) to 5 (*best*).

Burn scars (model 2) were evaluated before euthanasia using the modified Yeong scale (40), by two surgeons, blinded for the treatment groups. This three-item wound evaluation scale was specifically developed for thermal injuries assessing the scar surface appearance, height and color mismatch from 1 (best) to 4 (worst) for each item.

### Tissue Sampling

After blood collection, morphologic analysis was performed and captured using a 4K/12-megapixel camera. Next, the scar was dissected and evenly distributed into test tubes containing Bouin solution.

### Microscopic Grading

All specimen were evaluated histologically. In our burn model the scars were marked with blue dye for better microscopic evaluation and standardization. All specimen were then washed in phosphate buffered saline (PBS) and fixed in 10% buffered formalin before being embedded in paraffin and cut into 3 µm thick sections, slides were then stained using hematoxylin and eosin (H&E) and examined by two researchers who were blinded to the groups in light microscopy, using a magnification of ×4 and x10. Assessment of wound healing (epithelialization) was carried out in a standardized manner and expressed as a percentage of the whole wounded area. This was performed at a magnification of x10. The unhealed wound was measured as the distance between both edges of the reeptongue and the total wound diameter as the distance between the wound edges.

### Immunohistochemistry (H&E, Ly6g, Collagen I/III, SMA, and Fibrin)

Hematoxylin and Eosin (H&E) and Lymphocyte Antigen 6 Complex Locus G6D (1A8-Ly6G) staining was performed with a standardized staining procedure. Collagen fibers were stained using Pico Sirius red (ab150681, Abcam, Cambridge, UK), using polarized light microscopy was used to differentiate collagen I from III. An antibody for smooth muscle actin (SMA, ab5694, Abcam, Cambridge, UK) was applied to the samples, serving as a marker for myofibroblast, which induce wound contraction. Fibrin deposition was determined using a fibrinogen antibody (ab58207, Abcam, Cambridge, UK). Subsequently, the stained samples were incubated according to manufacturer’s instructions. In accordance with each antibody examined, an appropriate isotype control antibody was used as a negative control.

All samples were scored semi-quantitatively using following score:

- None (0) – no signs of tissue staining
- Isolated (1) – barely any staining of the tissue
- Little (2) – small amount of tissue staining
- Medium (3) – medium amount of tissue staining
- Strong (4) – strong amount of tissue staining

The assessment of collagen alignment was scored based on the orientation of the bundles (0=diffuse with bundles in 90° angle to 4=parallel).

### Immunofluorescence Staining (MPO, NE, and H3cit)

Three micrometer paraffin tissue sections underwent a deparaffinization and rehydration process followed by immunofluorescence staining for myeloperoxidase (MPO), neutrophil elastase (NE) and citrullinated histone 3 (H3cit). Antigen retrieval was assessed by incubating the sample slides with Target Retrieval Solution pH6 (Dako, Santa Clara, USA) and microwave for 1 min at 360W. following a cooling step of 30 min. After rinsing the sections twice for three min with a solution of tri-buffered saline and polysorbate 20 (Tween 20) (TBST), blocking of the probes was performed with a Donkey Block (BioGenex, Fremont, USA) for 30 min at room temperature (RT). Tissue specimens were further incubated with either isotype- or antigen-specific-antibodies at 4°C (Abcam, UK). Mouse anti-mouse MPO- (AB90810, Abcam, Cambridge, UK), rabbit anti-mouse NE- (AB68672, Abcam, Cambridge, UK), and rabbit anti-mouse H3cit- antibodies (AB5103, Abcam, Cambridge, UK) were diluted 1:50. Twelve hours later, sections were rinsed 3 x 5 min with TBST and subsequently incubated 1:200 with AF647- or Cy3 at RT for 30 min (Abcam, Cambridge, UK). After a 3 x 5 min rinsing-step with TBST, nuclei were counterstained by incubating probes with DAPI for 5 min at RT. Finally, slides were rinsed 5 min with PBS followed by 5 min rinsing with H_2_O, and mounted with Fluoromount-G (Southern Biotech, Birmingham, USA). Isotype control antibodies were used as a negative control (MPO = X0931, Aglient, Santa Clara, USA; NE = AB37415, H3cit = AB37415, Abcam, Cambridge, UK).

### Statistics

All data were analyzed using SPSS Statistics 26 (IBM, NY, USA) and GraphPad Prism 9 (GraphPad, CA, USA). A pre-power study calculation was performed using G*Power 3.0. The power was deducted from previous trials regarding inflammation and NET formation (13, 41). Differences between groups were calculated using mixed-effect model with Geisser-Greenhouse correction as well as Dunnett’s multiple comparison test. Data is presented as mean ± standard deviation (SD). The level of significance was set at 0.05.

## Results

All animals survived and no complications, such as wound infection, sepsis or incisional hernia occurred.

### Model 1—Laparotomy Model (Primary Intention Wound Healing)

#### Effects of Anti-NETs Treatment on Wound Healing

Treatment with DNase1 led to a significantly improved Stony Brook Scar Evaluation Scale at 21 days post wound induction compared to controls that received a vehicle. A similar effect occurred in the Pad4-KO mice, which scored significantly better in the scar scale in comparison to wild type mice treated with the vehicle (shown in **Figures 1A, B** **)**. With regards to immunohistochemistry, NETs seem to affect collagen distribution: There were no significant differences between groups regarding the collagen 1 to collagen 3 ratio, however, the alignment of collagen was significantly enhanced after 21 days post wound induction in mice treated with DNase1, compared to the control cohort (as shown in **Figures 1C–E**). Conversely, the collagen I to III ratio (p=0.012) and collagen alignment (p=0.03) of Pad4-KO were significantly increased in comparison to mice without endogenic DNase1 (DNase1-KO). Moreover, staining of fibrin was significantly reduced by DNase1 treatment (**Figure 1F**). SMA however, was not affected by treatment with DNASES or Pad-KO in this model (**Figure 1G**).

#### Effects of Anti-NETs Treatment of Neutrophils

**Figure 2:**
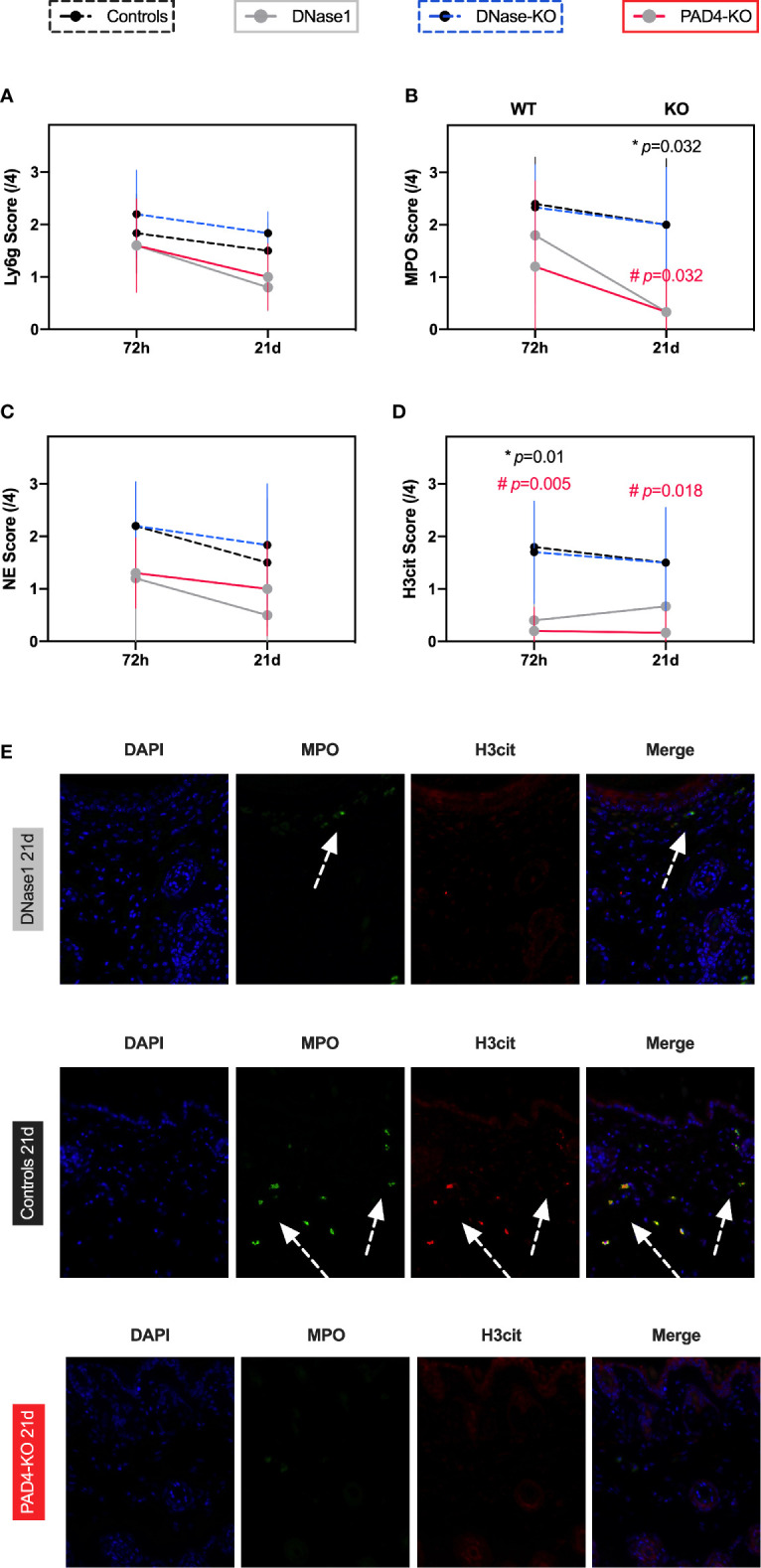
Therapeutic targeting of NETs formation results in decreased neutrophil activation and NETs formation in a model of primary intention wound healing. **(A)** Ly6G a marker of granulocytes was not affected by genetic alterations of NETs formation or DNase1 treatment. **(B–D)** Neutrophil activation and NETs formation was significantly reduced by DNase1 treatment or genetic knockout. **(E)** Representative immunofluorescence images. DNase1 treatment vastly reduced neutrophil activation (MPO) and NETs formation (H3cit). Data shown as Mean ± SD. Comparison was performed always in comparison with controls. Statistics: mixed-effect model with Geisser-Greenhouse correction as well as Dunnett’s multiple comparison test. *DNase1 vs. controls. ^#^PAD4-KO vs. controls.

Immunofluorescence staining showed that DNase1 treatment significantly reduced markers of neutrophil activation, in particular NE, and NETs formation (H3cit) compared to controls (**Figures 2B–E**). The effects were even more pronounced when comparing animals with limited NETs formation (Pad4-KO) to mice with a genetic knockout of DNase1 (MPO p=0.023, NE p=0.029, H3cit P=0.02, **Figures 2B–E**). In contrast, the effect of treatment with DNase1 or genetic alteration of NETs formation on neutrophils (Ly6G staining) was not significant (**Figure 2A**).

### Model 2: Thermal Injury (Secondary Intention Wound Healing)

#### Effects of Anti-NETs Treatment on Wound Healing

**Figure 3:**
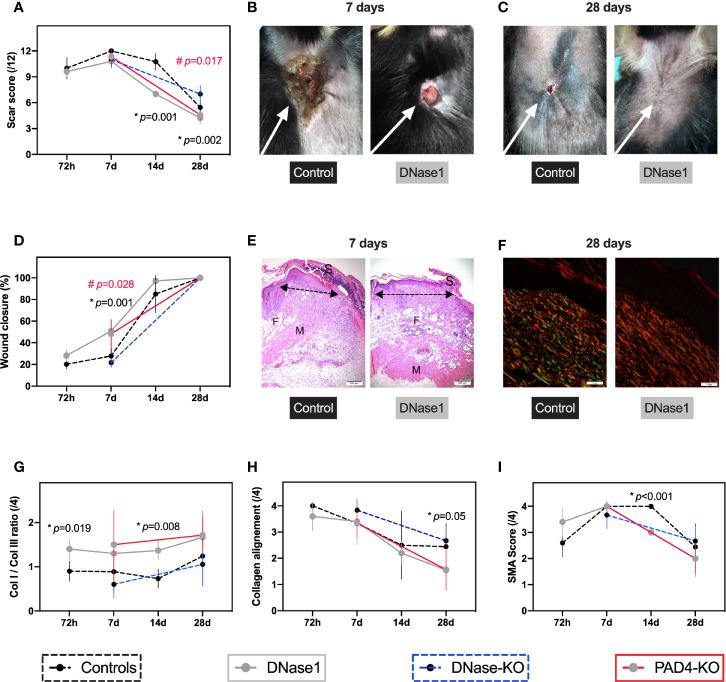
Therapeutic targeting of NETs improves secondary intention wound healing. In all animals a 1.5 cm^2^ thermal injury was induced. For the wildtype mice four timepoints (72 h, 7, 14, and 28 days) were performed and two (7, 28 days) for the knockouts. Controls n=5-6, DNase1 n=6, DNase1-KO n=5–6, Pad4-KO n=5–6. **(A–C)** Animals that received DNase1 or with limited NETs formation (Pad4-KO) had significantly superior scar scores than controls. **(D–H)** Animals with DNase1 treatment or without NETs showed a significantly faster wound closure and switch from collagen 3 to 1. Additionally, an improved collagen alignment after DNase1 treatment was found, indicating a faster maturation of the scar. **(E)** H&E staining of the thermal injury on day 7 showing the left wound border. Arrows indicate the reepithelization tongue progressing significantly faster in animals with DNase treatment. S indicates scab which is made up of necrotic tissue and found is less-optimal wounds. M indicates muscle layer and F adipose tissue. In control mice the subcutaneous fat and muscle layer was lost. However, in mice that were treated with DNase1 both layers were not affected possibly suggesting a secondary injury induced by extracellular traps. **(F)** Parallel collagen fiber formation was observed predominantly during early stages and persisted significantly in control and DNase1-KO scars until day 28; whereas basket wave-like texture reminiscent of normal skin was more evident in animals with DNase treatment or with limited NETs formation (Pad4-KO). Moreover, in controls and DNase-KO mice immature fibers (Collagen III; green vs. Collagen I, red) remained dominant. **(I)** SMA was only affected partially by DNase1. Data shown as Mean ± SD. Comparison was performed always in comparison with controls. Statistics: mixed-effect model with Geisser-Greenhouse correction as well as Dunnett’s multiple comparison test. *DNase1 vs. controls. ^#^PAD4-KO vs. controls.

Wound healing was very consistent in all animals but affected knockout mice differently: Treatment with DNase1 significantly improved scar appearance compared to controls as measured by the modified Yeong scar scale (**Figures 3A–C**). Additionally, mice with reduced NET concentration, either resulting from DNase1 treatment or due to the Pad4-KO, showed a significantly faster wound closure time compared to (untreated) controls or DNase1-KO as shown in **Figures 3D, E**. The acceleration of wound maturation in mice that received DNase1 for one week is reflected by a significantly improved collagen I to III ratio (**Figures 3F, G** **)** and collagen alignment (**Figures 3F, H** **)**. Collagen birefringence pattern was assessed. Parallel collagen fiber formation was observed predominantly during early stages and persisted significantly in control and DNase1-KO scars until day 28; whereas basket wave-like texture reminiscent of normal skin was more evident in animals with DNase treatment or with limited NETs formation (Pad4-KO). A statistically significant increased proportion of immature fibers (Collagen III; green) and decreased of mature fibers (Collagen I, red) were revealed in controls and DNase-KO animals. As in primary intention wound healing, SMA was mostly not affected by genetic alteration of NETs formation (Pad4-KO) or treatment with DNase1 (**Figure 3I**).

#### Effects of Anti-NETs Treatment of Neutrophils

**Figure 4:**
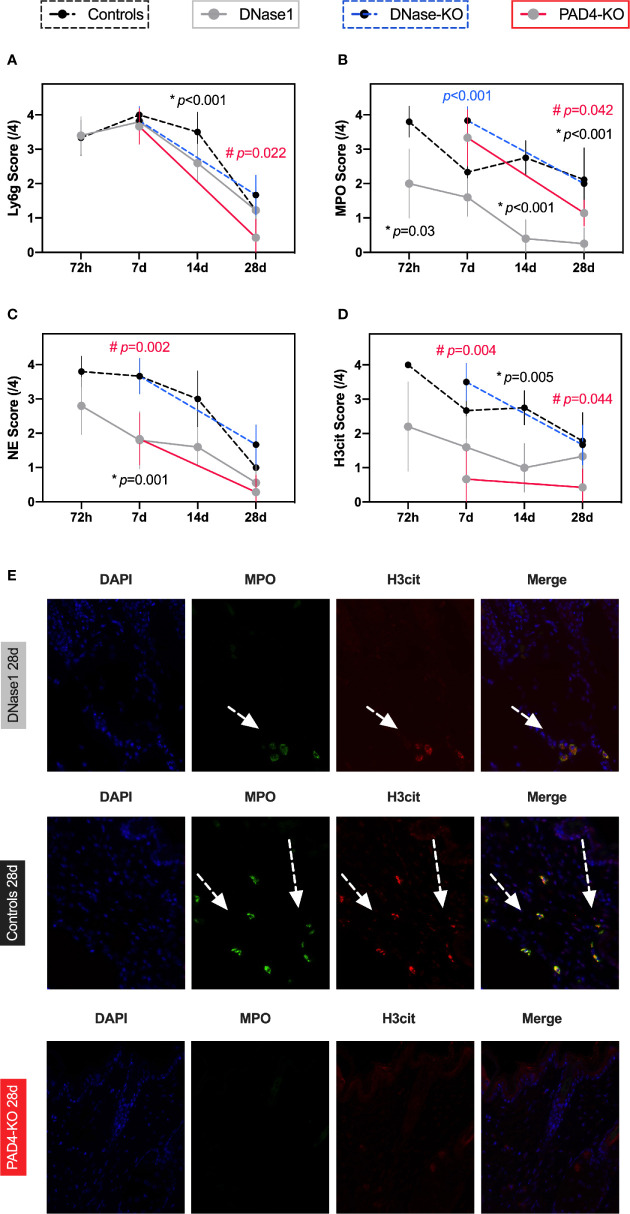
Therapeutic targeting of NETs reduces neutrophil activation and NETs formation in a model of secondary intention wound healing. **(A)** Comparable Ly6G levels indicate that granulocytes were not affected by DNase1 after thermal injury. However, in PAD4-KO mice a significant difference was found after 28 days, possibly indicating a loop effect of NETs and neutrophils. **(B, C)** Neutrophil activation remained high but was significantly lower in animals that received DNase1 or with limited NETs formation (Pad4-KO). **(D)** NETs formation can be significantly reduced by DNase1. **(E)** Representative immunofluorescence images. It appears that in burn wound neutrophils remain activated and produce NETs even after 4 weeks. DNase1 treatment is able to reduce this effect to some extent. Data shown as Mean ± SD. Comparison was performed always in comparison with controls. Statistics: mixed-effect model with Geisser-Greenhouse correction as well as Dunnett’s multiple comparison test. *DNase1 vs. controls. ^#^PAD4-KO vs. controls.

Compared to primary intention wound healing, inflammation remained high in the thermal injury model over time, as measured through neutrophil activation and NETs formation: DNase1 treatment did not significantly affect the number of neutrophils, however PAD4-mice showed a reduction of neutrophils after 4 weeks (**Figure 4A**). However, neutrophil activation (as measured by the MPO score and NE) and most importantly, NETs formation (as measured by H3cit) were significantly lower in animals that were treated with DNase1 compared controls (**Figures 4B–E**).

## Discussion

Wound healing is a complex biological process, and therapeutic enhancement has proven difficult. To date, various concepts to improve wound healing and to limit hypertrophic scaring exist, but these measures are complicated, time consuming, and relatively ineffective (42). Particularly after thermal injuries, 70% of all patients still suffer from hypertrophic scaring, despite continuous advances in the surgical management of burn injuries. Subsequently, quality of life is greatly decreased due to the massive functional, aesthetic, and psychosocial sequelae (43). In the current study, primary and especially secondary intention wound healing was improved significantly by targeting NETs either by DNase1 treatment or genetic knockout (Pad4-KO mice). Supporting these findings is the observation that the genetic knockout of DNase1 lead to a diminished wound healing. Thus, DNase1 treatment improved wound closure time and scar appearances, which were reflected by an improved collagen I to III ratio and collagen alignment. Fortunately, SMA, a marker of wound stability, was not affected by anti-NETs treatment.

The appearance of scar tissue is dependent on the diameter, density, and orientation of the collagen fibers within the wound (44). While collagen fibers in normal skin tissue show a basket-wave orientation, the fibers in scar tissue are densely packed and orientated in a parallel fashion (44, 45). Furthermore, the collagen fibrils appear to be thinner in contrast to normal tissue, which results in an altered ratio of collagen I to III (44). Therefore, it is believed, that the mechanical stability, the tensile strength, and the wound quality of both normal and scar tissue are determined by the ratio of collagen I to III and orientation (46, 47). In previous studies a low collagen I to III ratio has been associated with anastomotic leakage after large bowel surgery and with a higher incidence of incisional hernias and recurrent incisional hernias (46–48). In the current study, however, inhibition of NETs formation (by Pad4-KO or alternatively DNase1 treatment) lead to an improved collagen I to III ratio in the scars. Treating wounds with DNase1, one might fear an impairment of wound stability; especially in the context of thermal injuries in which neutrophils are known to persist for weeks (31). In contrast to this assumption, this study demonstrated that overall wound healing was not impaired, but rather accelerated, as shown by the improved switch from collagen III to I, the collagen alignment, and most obviously by the accelerated wound closure. Correspondingly, SMA concentrations did not differ between animals with increased or decreased NETs formation, which is expressed by the activated myofibroblast in the course of wound healing. Thus, the results may suggest that NETs do not promote the phenotype switch of fibroblast to myofibroblast (49).

In previous studies it has been established that neutrophils play an essential role in wound healing. Skin injury triggers neutrophil infiltration and NETs formation through unknown mechanisms (50). The increase in NET deposition in skin wounds of diabetic mice reduces healing rates and normal healing rates can be restored by PAD4 deficiency (3). The process might involve tissue damage or the modulation of inflammation and the downregulation of tissue repair mechanisms (51). In the current study, targeting of NETs by DNase1 or by the genetic knockout did not influence the number of neutrophils, but it did lead to a significant reduction of neutrophil activation and, most importantly, NETs deposition in the skin. The self-amplifying loop between activated neutrophils and NETs has been described in previous studies (52–57). As total number of neutrophils were not affected by DNase1 treatment, it appears that NETs affect neutrophil activation; for instance *via* an activation loop by oxidative stress or IL-1b/IL-18 (54, 56). Taking the results form Wong et al. under consideration, it is very likely that deposition of NETs in primary and secondary intention wound healing hinders wound healing and that this process may be improved using anti-NETs therapy like DNASES (3).

Limitations of the current study include (1) the limited number of timepoints of the knockout mice in the burn model and (2) the histological analyses are rather observational than providing mechanistic insights which should be addressed in future studies.

In conclusion, the inhibition of NETs formation either by treatment with DNase1 or Pad4-KO appears to be a promising option to reduce scar formation. Indeed, recombinant human DNase1 is cost effective and, to date, no adverse effects are known (58–61). In fact, NETs are shown to have procoagulant and prothrombotic effects, but as of yet no evidence for an elevated bleeding diathesis after NETs dissolution are known. In a previous study, systemic DNase1 treatment did not interfere with coagulation; more specifically, it did not affect bleeding time (41). Additionally, health care professionals might fear an elevated susceptibility to infections in those treated with DNase1, due to their role in the innate immune system. However, although NETs were first postulated to limit infection, a lack of NETs did not worsen bacteremia in PAD4-deficient mice which were subjected to polymicrobial sepsis, indicating that NET inhibition will not likely render the host vulnerable to bacterial infections (62). However, further research is necessary to validate our findings in humans and to test tolerances of the DNases in a clinical setting.

## Data Availability Statement

The original contributions presented in the study are included in the article/**Supplementary Material**. Further inquiries can be directed to the corresponding author.

## Ethics Statement

The animal study was reviewed and approved by Hamburg State Administration for animal research (73/15, 63/16).

## Author Contributions

AH: acquisition, analyzed data, and interpretation of the data, drafted the manuscript, and approved the final revision. CS: acquisition, analyzed and interpreted the data, drafted the manuscript, and approved the final revision. JE: acquisition, analyzed and interpreted the data, and approved the final revision. IK: acquisition, analyzed data, and approved the final revision. DV: acquisition, analyzed data, and approved the final revision. PS: acquisition, analyzed data, and approved the final revision. MT: acquisition, analyzed data, and approved the final revision. BA: acquisition, analyzed data, and approved the final revision. KR: acquisition, analyzed data, and approved the final revision. LP: acquisition, analyzed and interpreted the data, and approved the final revision. MB: designed study, acquisition, analyzed and interpreted the data, drafted the manuscript, and approved the final revision. All authors contributed to the article and approved the submitted version.

## Funding

This study was supported by the German Research Society (BO5534).

## Conflict of Interest

MB serves as a medical advisor of Neutrolis, Cambridge, MA, USA that focuses on developing therapies against NETs. MB is a stakeholder of Neutrolis. No compounds from Neutrolis were used in this study.

The remaining authors declare that the research was conducted in the absence of any commercial or financial relationships that could be construed as a potential conflict of interest.

## Supplementary Material

Supplementary Figure 1 Experimental design and treatment strategy. 1A: In mice either a laparotomy or thermal injury was induced. Treatment was started directly after induction. Subjects were treated according to their assigned group with DNase1 i.p. injections for three days (model one 1B) or one week (model two 1C). In order to control for secondary effects of the injection, controls received a vehicle.
