# Supplementary figures and images for: Therapeutic Targeting of Neutrophil Extracellular Traps Improves Primary and Secondary Intention Wound Healing in Mice
Source: Front Immunol. 2021 Feb 25;12:614347. doi: 10.3389/fimmu.2021.614347 (PMC7947714; doi:10.3389/fimmu.2021.614347)

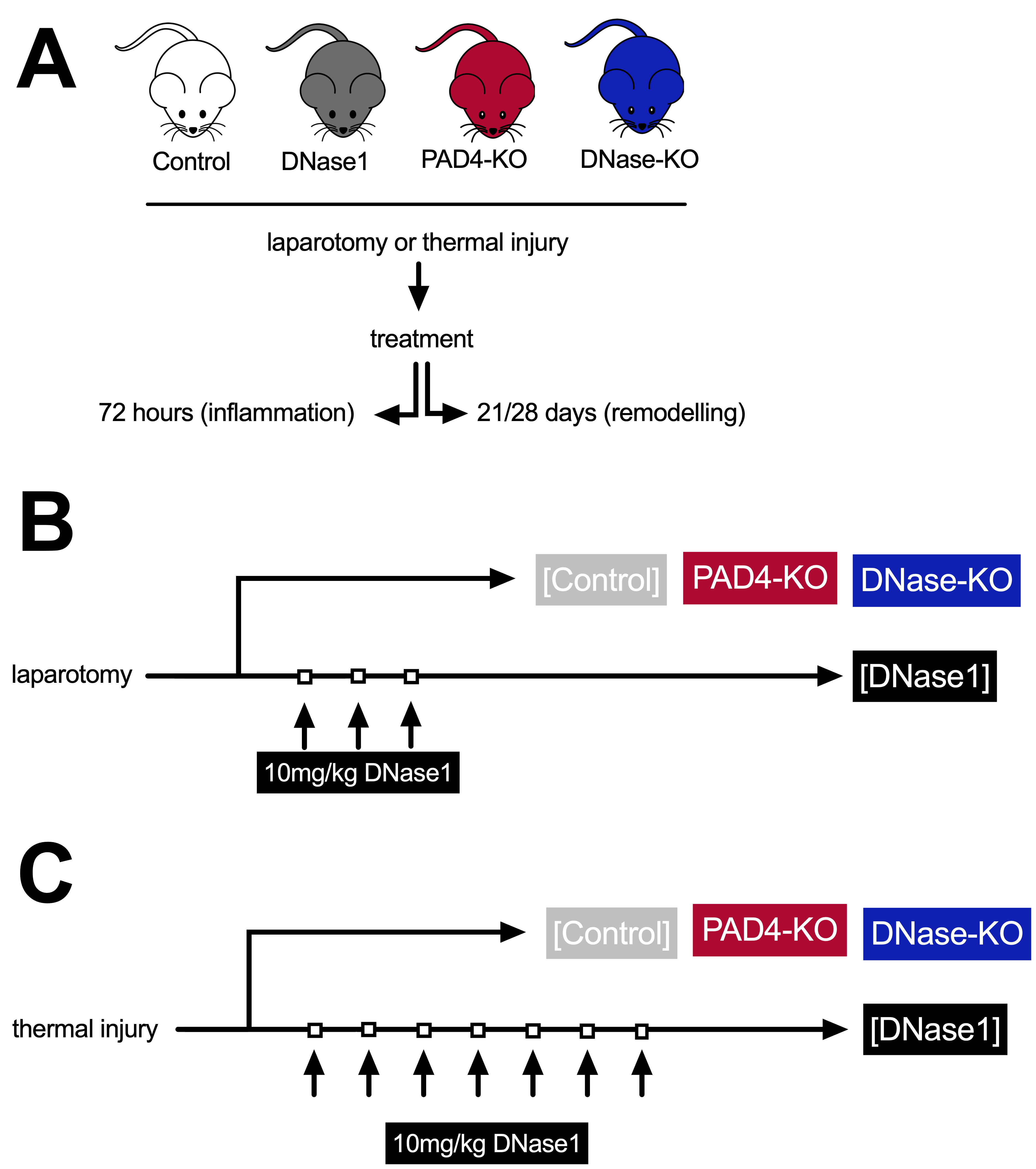

Supplement: Supplementary Figure 1 — Experimental design and treatment strategy. 1A: In mice either a laparotomy or thermal injury was induced. Treatment was started directly after induction. Subjects were treated according to their assigned group with DNase1 i.p. injections for three days (model one 1B) or one week (model two 1C). In order to control for secondary effects of the injection, controls received a vehicle. [file Image_1.tiff]
